# Response to two letters on omega-3 supplementation and cognitive decline

**DOI:** 10.1016/j.tjpad.2026.100662

**Published:** 2026-08-24

**Authors:** Zheng-Bin Liao, Ye-Ran Wang

**Affiliations:** a Department of Clinical Medicine, School of Basic Medicine, Third Military Medical University, Chongqing 400038, China; b Department of Rehabilitation, The Second Affiliated Hospital of Chongqing Medical University, No. 74 Linjiang Road, Yuzhong District, Chongqing 400010, China

Dear Editor,

We thank Huang, Lin, and Dong, and Dyall and Plourde, for their careful critiques of our article [1]. We emphasize that we did not claim a direct causal relationship between omega-3 supplementation and cognitive decline. As already stated in the published article, the retrospective observational design, exposure misclassification, and possible unmeasured confounding require cautious interpretation; the biological explanations were hypotheses, not established mechanisms. Nevertheless, the association across three cognitive measures, accompanied by longitudinal FDG change, warrants attention and independent confirmation. It is a signal for caution, not proof of harm or a basis for changing clinical practice. No new empirical analyses are reported here.

Exposure was derived from self-reported follow-up medication records, not a single baseline question. As explicitly stated in our published Discussion, precise dosage, discontinuation, and longitudinal adherence were unavailable, causing exposure misclassification. We also reported that the exposure included fish oil, flaxseed oil, and krill oil and that formulation-specific analyses were not feasible. Brand, exact composition, storage, and oxidation were additionally unavailable. Serum omega-3 concentrations were higher among users; the data were derived from serum samples analyzed by Nuclear Magnetic Resonance (NMR) on the Nightingale Health Platform, all samples were collected in the morning before breakfast and after an overnight fast (more details see: adni.loni.usc.edu). We therefore regard it as support for group classification, not validation of dose or long-term exposure. Continuous EPA+DHA exposure models were not performed. More complete, time-aligned measurements would be preferable [2].

The models included age, sex, APOE ε4 status, and diagnosis. As acknowledged, matching cannot exclude unmeasured confounding; we cited cardiovascular disease, family history of dementia, and chronic pain. Missingness further limits assessment of education, insomnia, and other confounders. We also stated that the pre-initiation analysis might miss subtle trajectories prompting supplementation. Thus, non-significant pre-initiation differences do not exclude reverse causality or confounding by indication. We identified no prespecified valid negative-control outcome and do not report conventional E-values because conversion of continuous slope differences to the risk-ratio scale requires additional assumptions [3].

The direction differs from Wei et al. [4], but the study estimated different quantities. Their 64% lower risk estimate concerned 68 long-term users with eight incident events; the fully adjusted HR was 0.37 (95% CI 0.18–0.75), versus 0.72 (0.54–0.97) for all users. Wei et al. excluded baseline dementia and assessed incident AD, whereas our broader sample assessed cognitive slopes after reported initiation. The discordance merits consideration, but neither analysis establishes causality. As emphasized in our study, inconsistent observational and trial evidence requires caution and further research. APOE ε4 was balanced between groups, although ADNI enrichment limits generalizability and does not address effect modification.

Figure 2 shows separate mixed-model fits, not observed within-person triplets of MMSE, ADAS-Cog13, and CDR-SB scores. The exposure-associated estimates are the between-group differences in annual slopes, not the total decline shown by the exposed-group lines. However, the curves extend to about 18 years despite a median follow-up of 5 years, without numbers at risk or observation density. Sparse late support, informative attrition, nonlinearity, and floor or ceiling effects therefore cannot be excluded, and terminal fitted values should not be interpreted as individual trajectories. The figure itself does not establish a scaling or harmonization error.

We have acknowledged in the manuscript that the longitudinal tau PET dataset was limited, reducing power to detect an association. The absence of detectable between-group differences in amyloid-beta, tau, or GMV slopes does not imply absent progression, but it constrains mechanistic interpretation. FDG SUVR is a non-specific relative index of cerebral FDG uptake, not a direct measure of glucose metabolism or synaptic injury [5]. The possible FDG pathway was a hypothesis; the product-of-coefficients result is a statistical indirect association, not proof of causal mediation. We did not perform partial-volume correction, alternative-reference analyses, or mediation using longitudinal synaptic or neuronal-injury biomarkers. Brain DHA, supplement oxidation, lipid-peroxidation products, and mitochondrial function were not measured; the oxidative mechanism therefore remains speculative.

Our findings do not justify stopping omega-3 supplements or changing clinical guidelines. However, absence of causal proof does not mean that the association should be disregarded. Within the limitations already discussed in the published article, reported use was associated with faster worsening across three cognitive measures and faster FDG SUVR decline. Alternative explanations remain, but the consistent signal warrants caution, clinical attention, and independent confirmation. Long-term randomized studies with well-characterized formulations, dose, adherence, baseline status, and prespecified biomarkers are needed.

## Declaration of competing interest

Ye-Ran Wang reports financial support from the 10.13039/501100001809National Natural Science Foundation of China, as disclosed in the original article. The other authors declare no known competing financial interests or personal relationships that could have influenced this response.

## Funding

The underlying study was supported by the 10.13039/501100001809National Natural Science Foundation of China (No. 82371435 to Ye-Ran Wang) and the Chongqing Municipal Health and Healthy Commission (Chongqing High-Level Medical Talent Program for Young and Middle-Aged Scholars, No. YXGD202506 to Ye-Ran Wang).

## Authorship/CRediT authorship contribution statement

The original manuscript was prepared by Dr. Zheng-Bin Liao and revised by Dr. Ye-Ran Wang.

## Declaration of the use of generative AI and AI-assisted technologies in scientific writing and in figures, images and artwork

Chat GPT5.5 was used to polish the language of the manuscript.

## CRediT authorship contribution statement

**Zheng-Bin Liao:** Writing – original draft. **Ye-Ran Wang:** Writing – review & editing.

## References

[1] Liao Z-B, Hu Z-C, Zeng G-H, Chen J., Li X-P, Liu Y-H (2026). The association between omega-3 supplementation and cognitive decline in older adults. J Prev Alzheimers Dis.

[2] Brenna J.T., Plourde M., Stark K.D., Jones P.J., Lin Y-H. (2018). Best practices for the design, laboratory analysis, and reporting of trials involving fatty acids. Am J Clin Nutr.

[3] VanderWeele T.J., Ding P. (2017). Sensitivity analysis in observational research: introducing the E-value. Ann Intern Med.

[4] Wei B-Z, Li L., Dong C-W, Tan C-C (2023). Alzheimer’s Disease Neuroimaging Initiative, Xu W. The relationship of omega-3 fatty acids with dementia and cognitive decline: evidence from prospective cohort studies of supplementation, dietary intake, and blood markers. Am J Clin Nutr.

[5] Stoessl A.J. (2017). Glucose utilization: still in the synapse. Nat Neurosci.

